# Cost Effectiveness of a Potential Vaccine for *Human papillomavirus*

**DOI:** 10.3201/eid0901.020168

**Published:** 2003-01

**Authors:** Gillian D. Sanders, Al V. Taira

**Affiliations:** * Stanford University, Stanford, California, USA

**Keywords:** Human papillomavirus, Vaccines, Cost-benefit analysis, Cervix neoplasms, research

## Abstract

*Human papillomavirus* (HPV) infection, usually a sexually transmitted disease, is a risk factor for cervical cancer. Given the substantial disease and death associated with HPV and cervical cancer, development of a prophylactic HPV vaccine is a public health priority. We evaluated the cost-effectiveness of vaccinating adolescent girls for high-risk HPV infections relative to current practice. A vaccine with a 75% probability of immunity against high-risk HPV infection resulted in a life-expectancy gain of 2.8 days or 4.0 quality-adjusted life days at a cost of $246 relative to current practice (incremental cost effectiveness of $22,755/quality-adjusted life year [QALY]). If all 12-year-old girls currently living in the United States were vaccinated, >1,300 deaths from cervical cancer would be averted during their lifetimes. Vaccination of girls against high-risk HPV is relatively cost effective even when vaccine efficacy is low. If the vaccine efficacy rate is 35%, the cost effectiveness increases to $52,398/QALY. Although gains in life expectancy may be modest at the individual level, population benefits are substantial.

Cervical cancer is one of the most common malignancies in women: this year in the United States, approximately 13,000 new cases will be diagnosed, and >4,000 women will die of the disease. Fortunately, cervical cancer is highly preventable with regular Papanicolaou (Pap) testing. Between 1973 and 1995, the Surveillance, Epidemiology, and End Results (SEER) Program (sponsored by the National Cancer Institute) documented a 43% decrease in incidence and a 46% decrease in death from cervical cancer. Such reductions, however, have not been observed in locations or countries where cytologic testing is not widely available. Epidemiologic research strongly implicates *Human papillomavirus* (HPV) as the major risk factor for cervical cancer. Therefore, methods of prevention, diagnosis, and treatment of HPV infection have been pinpointed as a means of reducing the incidence of cervical cancer.

HPV comprises >100 different types of viruses; approximately 40 of these are transmitted sexually. Although most HPV infections proceed and resolve without symptoms, some types of HPV (such as 6 and 11) may cause genital warts, whereas other types (such as 16 and 18) are associated with certain types of cancer. HPV infections are recognized as the major cause of cervical cancer: >90% of women who have cervical cancer also have been infected with HPV (*1*–*7*). HPV types that are correlated with the development of cancer are referred to as high-risk. Although no medical means currently exist to eliminate HPV infection, precancerous lesions and warts caused by these viruses can be treated.

Given the substantial disease and death associated with HPV and cervical cancer, research to develop a prophylactic HPV vaccine is ongoing (*8*). Vaccines for HPV-16 and HPV-18 are currently being studied in clinical trials; the Phase I trial results are encouraging (*9*,*10*). The cost effectiveness of such vaccines, however, has not been studied sufficiently. Therefore, we evaluated the effectiveness and cost effectiveness of a prophylactic vaccine.

## Data and Methods

**Figure 1:**
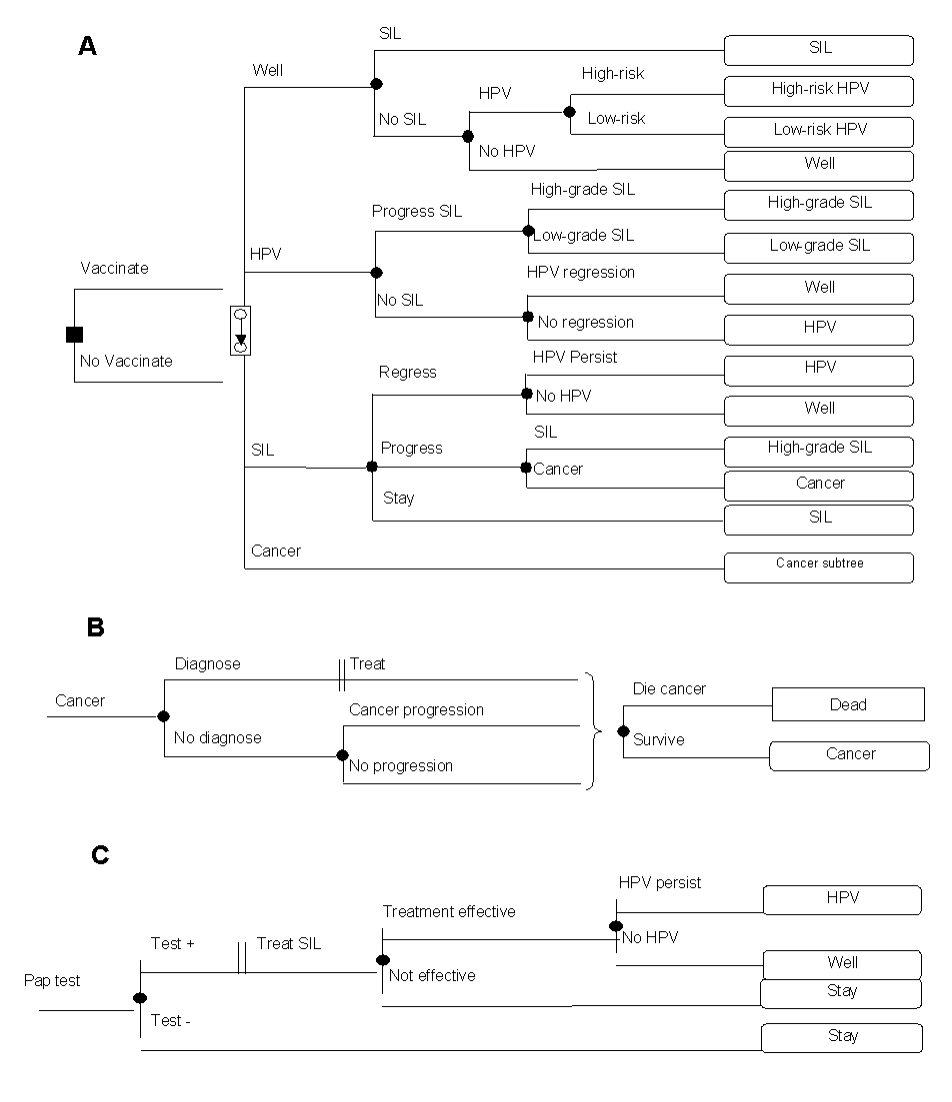
Schematic representation of the decision model. In panel A, the square node at the left represents the vaccination decision. The woman’s health thereafter is simulated by a Markov model. Each month, women are at risk of developing *Human papillomavirus* (HPV) infection, SIL (squamous intraepithelial lesions), or cervical cancer. Women who contract HPV may be infected by a low- or high-risk type. Panel B demonstrates cervical-cancer diagnosis, treatment, and natural history. Throughout a woman’s lifetime, her HPV, SIL, or cervical cancer status can be discovered either through development of symptoms or through routine Pap tests. Panel C shows that women who undergo a Pap test may test negative or positive for SIL.

We used a decision model to estimate the length of life and expenditures for vaccination of adolescent girls for high-risk HPV types (Figure 1). We adhered to the recommendations of the Panel on Cost Effectiveness in Health and Medicine (*11*) for conducting and reporting a reference-case analysis. We expressed our results in terms of costs, life-years, quality-adjusted life-years (QALYs), and incremental cost-effectiveness (ICE) ratios. We performed one-way sensitivity analyses on all model variables, as well as multi-way sensitivity analyses on selected variables.

### Patient Population

The target population for this analysis was all adolescent girls in the United States. Our base-case analysis considered a hypothetical cohort of 12-year-old girls. A recent study by the Centers for Disease Control and Prevention (CDC), indicated that although 3% of girls have had sexual intercourse before reaching age 13, 18.6% are sexually active by age 15, and 59.2% by age 18 (*12*). We therefore believed that vaccinating 12-year-old girls would capture most girls before they are sexually active and are at risk for HPV infection. We examined the optimal vaccination age in sensitivity analyses.

Our analysis assumes a universal vaccination strategy for adolescent girls. Although risk factors for HPV infection are identifiable, we chose to evaluate a universal vaccination program for several reasons. Previous vaccination programs aimed to reduce incidence of *Hepatitis B* *virus* (HBV) infection have tried to target the risk groups that account for most cases (*13*). These high-risk groups, however, are difficult to vaccinate for a variety of reasons, including inaccessibility, noncompliance, and the inability to identify people at risk. Also, because >30% of HBV-infected persons show no identifiable risk factor for infection (*13*,*14*), they would not be included in such a targeted immunization strategy. Similarly for HPV infection, the broad range of risk factors and the difficulty identifying these behaviors inhibit targeting such risk groups. We evaluated the cost-effectiveness of targeting high-risk girls (assuming a reduced compliance) in sensitivity analyses.

### Decision Model

We used Decision Maker software (Pratt Medical Group, Boston, MA, v2002.07.2) to develop a Markov model that followed the girls over their lifetimes. For each strategy, our model included probabilities of occurrence and progression of HPV, of squamous intraepithelial lesions (SIL), and of cervical cancer, as well as the probability of death, costs, and quality of life associated with the various health states. Whenever possible, we based our probability estimates (Appendix) on large, high-quality studies reported in the literature.

Our model (Figure 1A) tracks a cohort of girls who are either vaccinated against specific HPV types or who receive the current standard of care. Based on hepatitis B vaccination completion rates among U.S. adolescents, we assumed that 70% of the targeted girls would be vaccinated successfully (Appendix). We assumed that girls who were not vaccinated would receive the current standard of care.

Every month, each girl is at risk of developing high- or low-risk HPV, SIL, or cervical cancer. Over time, an infected woman’s HPV infection can regress, persist, or progress to either low- or high-grade SIL. SIL can also exist independent of an HPV infection. High-grade SIL can progress to cervical cancer. The diagnosis, treatment, and natural history of cervical cancer are modeled in Figure 1B.

We assumed that the current standard of care included routine Pap tests for compliant patients every 2 years starting at age 16. Throughout a woman’s lifetime, her HPV, SIL, or cervical cancer status can be discovered and treated either because symptoms have developed or through routine Pap tests (Figure 1C). We assumed that 10% of woman diagnosed with low-grade SIL would undergo cryotherapy and that all would receive a 6-week reexamination, and Pap tests at 3, 6, 12, and 18 months after cryotherapy. Treatment of high-grade SIL was assumed to include loop electrosurgical excision procedure (LEEP), and subsequent reexamination and Pap tests (*15*,*16*).

A woman may also choose to have a benign hysterectomy reducing her risk of cervical cancer. In addition to being at risk for death because of cervical cancer, all women are at risk for age-specific death unrelated to HPV or cervical cancer.

### Data and Base-Case Assumptions

#### HPV Infection

Incidence of HPV infection was based on Myers’ mathematical model of HPV infection (Appendix) (*17*). In our base-case analysis, annual incidence began at age 15 (10%), peaked at age 19 (18%), and dropped off quickly after age 29 (1%). We assumed that no prevalent HPV infections existed in the initial cohort of 12-year-old girls but varied this assumption in sensitivity analyses. Given HPV infection, regression rates were highest for women <25 years (46%/yr) and lowest for women >30 years (7%/yr), reflecting a preponderance of more persistent infections in the older age group (Appendix).

### Low- Versus High-Risk HPV

Because of a lack of significant HPV genotype cross-immunity, any vaccine developed probably will be effective against a limited number of HPV types (*18*,*19*). HPV types 16, 18, 45, and 31 together are the most commonly associated with cervical cancer, with evidence of these four types apparent in >75% of women who have cervical cancer (*20*). In our model, HPV types 16, 18, 31, 33, 35, 39, 45, 51, 52, 56, 58, 59, and 68 were considered high risk; all other HPV types were categorized as low risk (*20**–**22*). Based upon this classification in the general population, 59% of HPV infections are caused by high-risk types (Appendix).

### Low- and High-Risk Rates of HPV Progression

To evaluate potential vaccination strategies, we modeled different disease-progression rates in women infected with low- and high-risk HPV. By combining data on overall progression rates of HPV infection to cancer, with prevalence data on women infected with low- and high-risk HPV who had low- or high-grade SIL or cervical cancer, we estimated separate progression rates for low- and high-risk HPV infections. Data from seven articles were considered of high enough quality to be included in our analysis (*1*–*7*).

High-risk HPV infections were significantly more common in women who had cervical cancer than in women who had precursor lesions. Based on the results of seven studies (N=1609), high-risk HPV infection was detected in 56% of women who had low-grade SIL, in 83% of women who had high-grade SIL, and in 90% of women who had cervical cancer. Low-risk HPV infection was detected in 22%, 8%, and 3% of these women, respectively. No evidence of HPV infection was found in 22%, 9%, and 7% of these women, respectively (*1*–*7*). We then calculated relative progression rates for transition from high-risk, low-risk, or no HPV infection to low-grade SIL; from low-grade to high-grade SIL; and from high-grade SIL to cervical cancer (Appendix).

### Cancer Surveillance, Treatment, and Progression

We estimated that 71% of the adult female population received biennial Pap testing. Pap test sensitivity and specificity results were based on a meta-analysis conducted by the Duke Evidence-Based Practice Center (*17*,*23*). Diagnosis of asymptomatic cervical lesions depended on a woman’s likelihood of having a Pap test and on the sensitivity and specificity of this test.

Assessment of treatment effectiveness for cervical lesions was based on a review of 13 studies that detailed treatment effectiveness by lesion stage (Appendix). Initial treatment effectiveness was estimated at 97% and 94% for low-grade and high-grade SIL, respectively. Unsuccessful initial treatment of cervical lesions was followed with a repeat treatment (cryotherapy or LEEP) (77%), cone biopsy (18%), or hysterectomy (5%) (*24*), increasing treatment success. We based cancer progression rates, annual patient survival rates, and probability of symptoms by cancer stage on an analysis by Myers et al. (Appendix) (*17*). Myers et al. validated their data by comparing predicted distribution of cancer by stage for an unscreened population with data from studies of women who had had no prior screening.

### Benign Hysterectomy

We considered women who did not have cervical cancer but who had hysterectomies to be fully protected from cervical cancer. We tested this assumption in sensitivity analyses. Age-specific hysterectomy rates were based on data from the Hospital Discharge Survey of the National Center for Health Statistics (Appendix).

### HPV Vaccine Characteristics

In our model the HPV vaccine was administered by using a series of three injections in a school-based immunization program. Because vaccine longevity is uncertain, we assumed that successful vaccination conferred immunity for 10 years but that repeated booster shots every 10 years were required to maintain the vaccine’s efficacy. We evaluated the need for more frequent booster shots or a vaccine that conferred lifetime immunity in sensitivity analysis. For our base-case analysis, vaccine efficacy against high-risk HPV types was estimated at 75%. We tested the complete range of vaccine effectiveness (from 0% to 100%) because of the absence of efficacy data from Phase III clinical trials and because future marketed vaccines may target only a subset of the high-risk HPV types.

### Quality of Life

HPV infection and cervical cancer can markedly affect quality of life and therefore can affect a woman’s quality-adjusted life expectancy. Accordingly, we incorporated adjustments for quality of life associated with current health, HPV, SIL, and with cervical cancer and its treatment.

Utilities for health states were based on a report by the Institute of Medicine on Vaccines for the 21st Century, which used committee-consensus Health Utility Indices levels for relevant health states (Appendix). Undiagnosed HPV and cervical lesions were considered to be asymptomatic and to have no utility decrement. Diagnosed and treated low- and high-grade SIL were assigned lower utilities (0.97) for a 1-year duration. Treatment for locally invasive cancer was assigned a low utility (0.79–0.80) during 4 months of initial treatment, with a moderate utility (0.90–0.97) during a 2- or 3-year follow-up. For more advanced cancer, a woman’s utility was decreased to 0.62 during both treatment and follow-up to reflect the severity of her disease and its effects on quality of life. We based current health utilities on the gender- and age-specific data from the Beaver Dam study (*25*).

### Costs

We converted all costs to 2001 U.S. dollars by using the gross-domestic-product deflator. Pap-testing costs were $81 per test, including a 10% rescreen rate. We estimated the cost of the vaccine materials, personnel, and administration at $300, based on school-based HBV vaccination programs (Appendix). We assumed a three-injection protocol with a booster shot ($100) required every 10 years.

Treatment costs of low- and high-grade SIL were based on Medicare average reimbursements and resource-based cost estimates. We estimated the cost of treatment of low-grade SIL from the cost of an initial colposcopy and biopsy, cryotherapy (in 10% of patients), a 6-week reexamination, and Pap tests at 3, 6, 12, and 18 months after treatment. The cost of treatment of high-grade SIL was based on cost of initial colposcopy and biopsy, LEEP, and subsequent reexamination and Pap tests. Cost of cancer treatment varied, depending on the stage at which cancer was diagnosed. Costs were based on Medicare average reimbursement rates (*26*) and cross-checked with a 1999 HMO case-control full-cost analysis (*27*) (Appendix).

### Sensitivity Analysis

We performed one-way and multi-way sensitivity analyses to account for important model uncertainties. For clinical variables, our ranges for sensitivity analyses represent our judgment of the variation likely to be encountered in clinical practice, based on the literature and on discussion with experts. The ranges for costs represent variation by 25% above and below the base-case estimate. To determine ranges for utilities, we used clinical judgment.

## Results

### Model Validation

We evaluated outcomes in the current practice arm of the model to ensure that they reflected the frequency of events from the Surveillance, Epidemiology and End Results (SEER) registry. Our model’s annual rates of cervical-cancer cases and cervical-cancer-related deaths match 2001 SEER estimates, as well as those calculated by the Myers model (*17*) (data available from the authors).

### Base-Case Analysis

A prophylactic vaccine against high-risk HPV types is more expensive than current practice but results in greater quality-adjusted life expectancy (Table 1). HPV vaccination of 12-year-old girls improves their life expectancy by 2.8 days or 4.0 quality-adjusted life days at a cost of $246 relative to current practice (ICE of $22,755/QALY).

**Table 1:** Health and economic outcomes of HPV vaccination^a^

Outcome	No vaccination	HPV vaccination
Cost, $	39,682	39,928
Incremental cost, $		246
Life expectancy, yrs	28.785	28.793
Incremental life expectancy, days		2.8
Quality-adjusted life expectancy, yrs	27.720	27.731
Incremental quality-adjusted life expectancy, days		4.0
Incremental cost effectiveness		
$/life year		32,066
$/quality-adjusted life year		22,755

^a^HPV, *Human papillomavirus*.

Vaccinating the present U.S. cohort of 12-year-old girls (population approximately 1,988,600) averts >224,255 cases of HPV, 112,710 cases of SIL, 3,317 cases of cervical cancer, and 1,340 cervical-cancer deaths over the cohort’s lifetime. Prevention of one case of cervical cancer would require vaccination of 600 girls (Table 2).

**Table 2:** Intermediate health outcomes of HPV vaccination^a,b^

Outcome	HPV vaccination	No vaccination	Lifetime cases averted	No. needed to vaccinate to prevent one case
HPV	1,460,699	1,684,954	224,255	9
SIL	417,549	530,259	112,710	18
Cervical cancer	13,374	16,690	3,316	600
Cervical-cancer deaths	5,121	6,461	1,340	1,484

^a^Assumes program that successfully administers a vaccine against high-risk HPV to the current U.S. cohort of 12-year-old girls.

^b^HPV, *Human papillomavirus*; SIL, squamous intraepithelial lesions.

### Sensitivity Analyses

**Figure 2:**
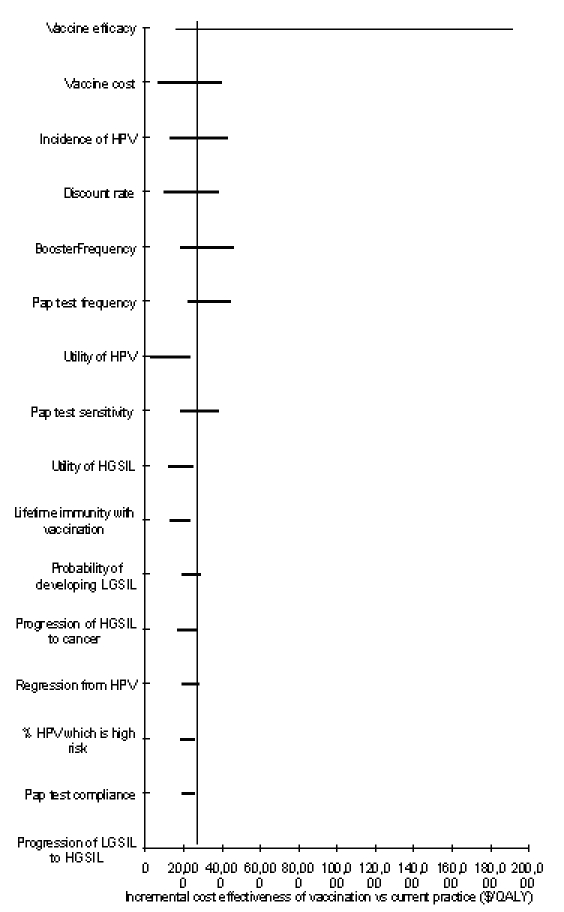
Sensitivity analysis. Tornado diagram representing the incremental cost-effectiveness ratios of one-way sensitivity analysis on the vaccination strategy compared to current practice. The vertical line represents the incremental cost-effectiveness ratio under base-case conditions.

Figure 2 shows the ICE ratios of one-way sensitivity analyses of the vaccination strategy compared to current practice. We explored those variables with the greatest effect on the ICE ratio by running more extensive sensitivity analyses. Given the uncertainty surrounding the vaccine efficacy, pricing, and mechanism, we performed extensive sensitivity analyses using vaccine-related variables.

**Figure 3:**
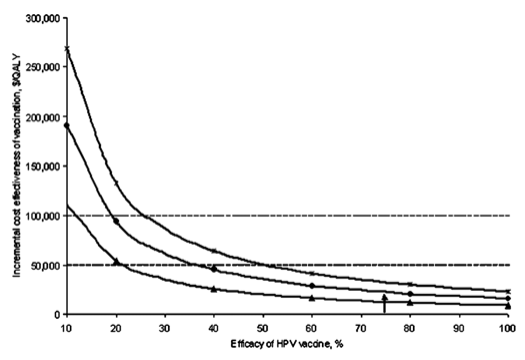
Sensitivity analysis: Vaccine efficacy. Effect of a change in *Human papillomavirus* (HPV) vaccine efficacy on the cost effectiveness of vaccination compared with current practice under varying assumptions of vaccine immunity. The triangles represent a vaccine which provides lifetime immunity to high-risk types of HPV. The circles represent a vaccine which requires booster shots every 10 years to remain effective (base-case assumption). The hatches represent a vaccine that requires booster shots every 5 years to remain effective. The dashed line indicates the $50,000 per quality-adjusted life year cost-effectiveness threshold. The base-case value of 75% efficacy is indicated by the arrow.

**Figure 4:**
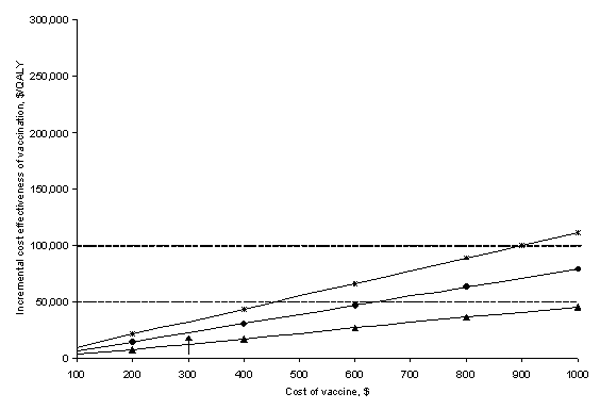
Sensitivity analysis: Vaccine cost. Effect of a change in *Human papillomavirus* (HPV) vaccine cost on the cost effectiveness of vaccination compared with current practice under varying assumptions of vaccine immunity. The triangles represent a vaccine that provides lifetime immunity to high-risk types of HPV. The circles represent a vaccine that requires booster shots every 10 years to remain effective (base-case assumption). The hatches represent a vaccine that requires booster shots every 5 years to remain effective. The dashed line indicates the $50,000 per quality-adjusted life year cost-effectiveness threshold. The base-case value of $300 is indicated by the arrow.

In our base-case analysis, we estimated that an HPV vaccine would provide immunity against high-risk HPV types in 75% of the girls vaccinated. At early stages of vaccine development or given a vaccine that targets only selected high-risk HPV types, the efficacy may prove to be lower. Sensitivity analyses on the vaccine efficacy and cost showed that even if the efficacy was reduced to 40% or the vaccine cost was increased to $600, vaccination costs <$50,000/QALY, relative to current practice (Figures 3 and 4).

We assumed that vaccination required a one-shot booster every 10 years. We also considered that vaccination could provide lifetime immunity, in which case the ICE improved to $12,682/QALY. Vaccinating the present U.S. cohort of 12-year-old girls with such a lifetime vaccine would avert >272,740 cases of HPV, 174,208 cases of SIL, 7,992 cases of cervical cancer, and 3,093 cervical-cancer deaths over the cohort’s lifetime. Prevention of one case of cervical cancer would require vaccination of 250 girls. Even if a booster shot is required every 3 years, the vaccine compared to current practice remained fairly cost effective ($45,599/QALY) (Figures 3 and 4). Our model assumes that a vaccination program would target 12-year-old girls for vaccination. Waiting until girls are 15 years old to provide vaccination results in a slightly lower life expectancy (reducing quality-adjusted life expectancy by 0.2 days) though at a reduced cost ($20). Vaccination of 12-year-old girls as compared to 15-year-old girls costs $40,440 per additional quality-adjusted life year gained.

**Figure 5:**
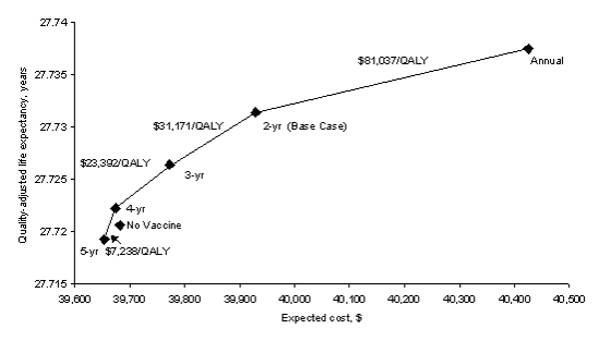
Sensitivity analysis: Frequency of Pap tests in vaccinated women. Effect of changing the frequency with which vaccinated women receive a Pap test. The diamonds represent Pap testing vaccinated women annually, every 2 years (base case), every 3 years, every 4 years, and every 5 years. The x-axis represents the lifetime expected cost of the vaccination strategy; the y-axis is the quality-adjusted life expectancy in years. The incremental cost effectiveness of increasing the frequency of Pap testing for vaccinated women is indicated numerically above the cost-effectiveness frontier.

Although the estimates used in our analysis reflect current Pap-test characteristics and compliance, if every woman obtained a Pap test every 2 years (base-case estimate is 71% compliance every 2 years), the ICE of vaccination increases to $33,218/QALY. Our base-case analysis assumes that vaccinated women would continue to receive Pap tests at the same frequency as unvaccinated women. HPV vaccination and the resulting reduction in cervical-cancer risk, however, might decrease frequency of Pap testing. Figure 5 shows how the costs and quality-adjusted life expectancy are influenced by the frequency of Pap tests in the vaccinated cohort. A strategy in which vaccinated women have Pap testing every 4 years increases life expectancy while reducing costs compared to current practice. While providing more frequent Pap tests to vaccinated women does increase a woman’s quality-adjusted life expectancy, it also increases costs. The cost-effectiveness ratios of more frequent testing are shown in Figure 5.

Our results were sensitive to several of our base-case assumptions (Figure 2). Vaccination saved 11.4 quality-adjusted life days and cost $290 over current practice when costs and benefits were not discounted (ICE of $9,286/QALY). At a discount rate of 5%, vaccination cost $37,752/QALY. Some women may be quite alarmed by being diagnosed with high-grade SIL; decreasing the utility of high-grade SIL to 0.8 lowers the cost-effectiveness ratio to $16,927/QALY. Varying the underlying incidence of HPV from 0.5 to 2 times our base-case values resulted in cost-effectiveness ratios ranging from $43,088 to $12,664 per QALY, respectively. Sensitivity analyses with other variables did not change our results substantially (Figure 2).

## Discussion

We evaluated the usefulness of a potential vaccine against high-risk HPV types administered to adolescent girls and found it to be cost effective as compared to current practice ($22,755/QALY). Although the increase in quality-adjusted life expectancy from a vaccination program is modest for the individual, the increase aggregates to substantial numbers of HPV infections, cases of cervical cancer, and prevented cancer-related deaths (Table 2). Furthermore, the life-expectancy gains are similar to those realized by current vaccination programs. Vaccination against high-risk HPV saved 2.8 life days and 4.0 quality-adjusted life days per person. In comparison, vaccinations against measles, mumps, rubella, and pertussis each save 2.7, 3.0, 0.3, and 3.3 life days, respectively (*28*,*29*). Sensitivity analyses found that the HPV vaccine would be cost effective, even assuming vaccine efficacy as low as 40% or that booster shots would be required every 3 years.

The only previous analysis of the cost effectiveness of a vaccine against HPV was published by the Institute of Medicine (IOM) (*30*). That analysis also showed an HPV vaccine to be cost effective. Our analysis differs from the IOM’s, however, in that we modeled a vaccine specific to high-risk types of HPV because such vaccines are under development and in clinical trials. In addition, our progression and recurrence rates are HPV-type specific.

Our analysis does have limitations. We analyzed the benefits and costs of vaccinating only adolescent girls against HPV. Because HPV is sexually transmitted, reducing the prevalence of HPV in the population will also affect the prevalence of HPV in women’s sexual partners. Although HPV is most commonly associated with cervical cancer, it may also play a role in cancers of the anus, vulva, vagina, and penis. The benefits of HPV vaccination associated with reductions in these types of cancers are not included in our analysis. Including them should make HPV vaccination even more favorable. The decision whether to vaccinate adolescent boys as well is more complex; therefore, in future work we plan to extend our analysis to incorporate such costs and benefits. In addition, the costs and benefits used in this analysis are tailored to the population and health-care environment of the United States. As Figure 5 demonstrates, the availability of HPV vaccines may justify less frequent Pap tests. This effect may be particularly relevant in developing countries that must decide how best to allocate their limited health-care resources.

We make several assumptions about the target vaccination population and program implementation that need discussion in terms of their political and social feasibility. First, we propose a school-based vaccination program rather than a clinic-based one. School-based immunization programs address several challenges encountered when vaccinating adolescents. First, school-based programs provide an infrastructure in which to vaccinate adolescents. Adolescent health-care visits are often not routine, and given scheduled visits, adolescents are often noncompliant with appointments. In addition, we believe that fitting the three-dose HPV vaccination regimen into the academic year will increase compliance while containing costs. Several school-based programs have documented completion rates of >90%. In contrast, lower rates of completion (11% to 87%) have been found in more traditional health-care settings (*31*–*34*). Second, we propose providing universal vaccination rather than targeting specific high-risk groups. Certain groups of women are at higher risk for HPV infection, and the cost effectiveness of vaccinating such target groups may be more favorable than a universal vaccination program. Experience with *Hepatitis B* vaccination in adolescents, however, has demonstrated how such groups may be those that are hardest to reach (*13*,*14*,*35*), and that many risk factors for infection (such as number of partners) may not be readily identifiable (*13*,*14*). Finally, we propose vaccinating girls at an early adolescent age (12 years). Although the lifetime cost of vaccinating 12-year-old girls is slightly greater than that of vaccinating 15-year-old girls, earlier vaccination costs <$50,000 per QALY when compared to costs of vaccinating older adolescents. A significant proportion of adolescents are sexually active by 15 years of age; therefore, vaccination at 12 years of age aims to include as many girls as possible before sexual activity begins and HPV infection risk increases. In addition, studies using *Hepatitis B* vaccines as a proxy have found better immune responses in younger persons and have shown that younger children require lower doses (*36*,*37*). Finally, we believe a 3-dose school-based vaccination program aimed at 12-year olds will result in greater compliance because adolescents of this age have more consistent school attendance (*13*,*38*–*40*). Before a HPV vaccination program is successfully implemented, social and political issues will need to be addressed and agreed upon by stakeholder groups, including pediatricians, public health officers, parents, adolescents, school administrators, and community leaders.

Several institutions, including Merck Research Laboratories, MedImmune Inc., GlaxoSmithKline, and the National Cancer Institute (NCI), are developing and testing prophylactic HPV vaccines. Researchers at NCI and Johns Hopkins have developed a virus-like particle vaccine with promising initial results (*9*,*10*). If the results of the recently completed Phase II study in the United States and Costa Rica confirm the Phase I results, a Phase III trial in Costa Rica involving 10,000 women will begin. Nonetheless, a vaccine probably will not be approved for widespread use for 3–5 years. Meanwhile, the need for continued cervical-cancer screening and treatment programs remains high.

Our study suggests that vaccination of girls with a HPV vaccine is cost effective when compared to many other generally acceptable health interventions. Although HPV vaccines are still under development, our assessment of the cost effectiveness, however, is robust across a wide range of vaccine mechanisms and efficacies. Although several hurdles to an HPV vaccine must be overcome before it is widely disseminated, our analysis suggests that a vaccine against high-risk HPV would have substantial public health benefit and emphasizes the importance of ongoing vaccine research and development.

## Supplementary Material

Appendix Table Input variables and sourcesa
