# Supplementary material for: Cost Effectiveness of a Potential Vaccine for Human papillomavirus
Source: Emerg Infect Dis. 2003 Jan;9(1):37–48. doi: 10.3201/eid0901.020168 (PMC2873748; doi:10.3201/eid0901.020168)
Supplement: Appendix Table — Input variables and sourcesa [file 02-0168_appT-s1.pdf]

Appendix Table. Input variables and sources[a]

Input variable	Base-case estimate	Range	Source
Population starting age, y	12	0–25	Assumed
**Vaccine variables**			
Vaccine effectiveness, %	75	0–100	Estimate
Vaccine compliance, %	70	30–100	(1,2)
Booster shot frequency, y	10	3–lifetime	Assumed
**Treatment variables**			
Initial treatment efficacy, given high-grade SIL, %	95	88–97	(3–6)
Treatment efficacy (including retreat), given high-grade SIL, %	99.5	99–100	(3–7)
Probability HPV infection persists, given effective treatment of high-grade SIL	10	0–25	Assumed
Initial treatment efficacy, given low-grade SIL, %	98	93–100	(8–10)
Treatment efficacy (including retreatment), given low-grade SIL, %	99.5	99–100	(7–10)
Probability HPV infection persists, given effective treatment of low-grade SIL	10	0–25	Assumed
**Surveillance variables**			
Pap test sensitivity for SIL (both low- and high-grade)	51	40–80	(11,12)
Pap test specificity for SIL (both low- and high-grade)	97	95–98	(11,12)
Compliance with Pap testing, %	71	60–80	(13,14)
Pap testing frequency in unvaccinated population, months	24	12–60	(13,14)
Pap testing frequency in vaccinated population, months	24	12–60	(13,14)
**HPV variables**			
Prevalence of HPV in initial cohort population, %	0	0–25	(12,15)
Annual incidence of HPV infection, given woman aged (yrs):		0.5–2x	(12)
0–15	0		
15–16	0.1		
17	0.12		
18	0.15		
19	0.17		

20	0.15		
21	0.12		
22–23	0.10		
24–29	0.05		
30–49	0.01		
50+	0.005		
Proportion of high-risk HPV infections, %[a]	59	52–72	(16–19)
Annual probability (%) of HPV infection resolving, woman aged (yrs):			(12,18,20,21)
0–24	45.7	40–55	
25–29	32.9	30–37	
30+	6.8	4–10	
**SIL variables**			
Annual probability of SIL, given no HPV infection, %	0.025	0.02–0.03	(22–26)
Annual probability of SIL, given low-risk HPV infection, %	3.6	3–5	(12,14,22–27)
Annual probability of SIL, given high-risk HPV infections, %	6.5	5–8	(12,14,22–27)
Low-grade SIL, given no HPV infection, %	100	90–100	Assumed
Low-grade SIL, given HPV infection, %	90	80–100	(12)
Annual probability (%) of low-grade SIL regressing, given woman aged (yrs):			(27–36)
0–34	14.2	12–16	
35–44	5.8	4–8	
45+	2.7	2–8	
Probability of low-grade SIL regressing to previous state of HPV infection, given regression occurs, %	10	0–20	(12)
Annual probability (%) of high-grade SIL regressing, given woman aged (yrs):			(27,28,30,31)
0–44	5.8	3–7	

45+	3.7	3–7	
Probability of high-grade SIL regressing to well state, given regression, %	45	40–50	(12)
Probability of high-grade SIL regressing to previous state of HPV infection, given regression, %	5	0–10	Assumed
Probability of high-grade SIL regressing to low-grade SIL given regression, %	50	40–60	(12)
Annual probability (%) of developing high-grade SIL from low-grade SIL with no HPV infection, women aged (yrs):			(12,14,22–27)
0–34	0.5	0.3–0.7	
35–44	3.1	2–5	
45+	4.5	3–6	
Annual probability (%) of developing high-grade SIL from low-grade SIL when low-risk HPV infection is present, women aged (yrs):			(12,14,22–27)
0–34	0.4	0.2–0.6	
35–44	2.7	2–4	
45+	3.8	3–5	
Annual probability (%) of developing high-grade SIL from low-grade SIL when high-risk HPV infection is present, women aged (yrs):			(12,14,22–27)
0–34	2.0	1–3	
35–44	15.6	7–20	
45+	31.3	15–35	
Annual probability of developing cervical cancer, given high-grade SIL and no HPV infection, %	2.6	2–4	(12,14,22–27)
Annual probability of developing cervical cancer, given high-grade SIL developed through low-risk HPV infection, %	1.0	0.7–1.5	(12,14,22–27)
Annual probability of developing cervical cancer, given high-grade SIL developed through high-risk HPV infection, %	3.8	3–6	(12,14,22–27)
**Cervical cancer variables**			
Annual probability of progressing from undiagnosed Stage I cervical cancer to Stage II	43.7	40–45	(12)

cervical cancer, %			
Annual probability of progressing from undiagnosed Stage II cervical cancer to Stage III cervical cancer, %	53.5	50–55	
Annual probability of progressing from undiagnosed Stage III cervical cancer to Stage IV cervical cancer, %	68.3	65–70	
Annual probability of symptoms with undiagnosed Stage I cervical cancer, %	15	12–18	
Annual probability of symptoms with undiagnosed Stage II cervical cancer, %	22.5	20–25	
Annual probability of symptoms with undiagnosed Stage III cervical cancer, %	60	67–73	
Annual probability of symptoms with undiagnosed Stage IV cervical cancer, %	90	87–93	
Annual probability of survival after diagnosis, by stage:			(12)
Stage I			
Year 1	0.9688	0.95–0.99	
Year 2	0.9525	0.93–0.97	
Year 3	0.9544	0.93–0.97	
Year 4	0.9760	0.95–0.99	
Year 5	0.9761	0.95–0.99	
Stage II			
Year 1	0.9066	0.88–0.92	
Year 2	0.8760	0.85–0.89	
Year 3	0.9225	0.90–0.94	
Year 4	0.9332	0.91–0.95	
Year 5	0.9604	0.94–0.98	
Stage III			
Year 1	0.7064	0.68–0.72	
Year 2	0.7378	0.71–0.75	

Year 3	0.8610	0.84–0.88	
Year 4	0.9231	0.90–0.94	
Year 5	0.9142	0.89–0.93	
Stage IV			
Year 1	0.3986	0.37–0.41	
Year 2	0.4982	0.47–0.51	
Year 3	0.7638	0.74–0.78	
Year 4	0.8652	0.84–0.88	
Year 5	0.8592	0.83–0.87	
Time to remission, yrs	5		
Five-year survival after diagnosis, by stage, %			(12)
Stage I	83.9		
Stage II	65.66		
Stage III	37.87		
Stage IV	11.27		
**Costs, $**			
Vaccine	300	100–500	(37–40)
Booster shot	100	30–130	Assumed
Cost of treatment for cervical cancer, Stage I	14,979	11,234–18,724	(41,42)
Cost of treatment for cervical cancer, Stage II	21,811	16,358–27,264	(41,42)
Cost of treatment for cervical cancer, Stage III	21,811	16,358–27,264	(41,42)
Cost of treatment for cervical cancer, Stage IV	24,004	18,003–30,005	(41,42)
Cost of Pap test (w/10% retest)	81	61–101	(42)
Cost of treatment for high-grade SIL	1,218	914–1523	(42–45)
Cost of treatment for low-grade SIL	630	473–788	(42–45)

Cost of treatment for a false-positive SIL	230	172–288	(43,44,46)
Cost of hysterectomy	7,883	5912–9854	(11,45)
**Annual probability of hysterectomy by age (yrs), %**		0.25–2x	(47)
15–24	0.04		
25–29	0.35		
30–34	0.60		
35–39	0.99		
40–44	1.29		
45–54	0.99		
≥55	0.33		
**Utilities**			(48)
Low-grade SIL	0.97	0.8–1	
High-grade SIL	0.97	0.5–1	
Low-risk HPV infection	1.00	0.9–1	
High-risk HPV infection	1.00	0.8–1	
Cervical cancer, treatment phase			
Stage I	0.79	0.25–1	
Stages II–IV	0.62	0.25–1	
Cervical cancer, follow-up			
Stage I	0.90	0.25–1	
Stages II–IV	0.62	0.25–1	
Well	1.00		Age-specific utilities based on (49)
**Other Variables**			
Markov model cycle length, months	1		Assumed
Discount rate, %	3	0–5	(50)

[a]HPV, *Human papillomavirus*; SIL, squamous intraepithelial lesion. High risk HPV is defined as HPV 16, 18, 31, 33, 35, 39, 45, 51, 52, 56, 58, 59, 68; low-risk HPV is defined as all other types. All probabilities are annual unless otherwise noted. All costs are in 2001 U.S. dollars.

Appendix References

1. Yusuf H, Daniels D, Mast EE, Coronado V. Hepatitis B vaccination coverage among United States children. Pediatr Infect Dis J 2001;20(11 Suppl):S30–3.
2. Middleman AB, Robertson LM, Young C, Durant RH, Emans SJ. Predictors of time to completion of the hepatitis B vaccination series among adolescents. J Adolesc Health 1999;25:323–7.
3. Gunasekera PC, Phipps JH, Lewis BV. Large loop excision of the transformation zone (LLETZ) compared to carbon dioxide laser in the treatment of CIN: a superior mode of treatment. Br J Obstet Gynaecol 1990;97:995–8.
4. Oyesanya OA, Amerasinghe CN, Manning EA. Outpatient excisional management of cervical intraepithelial neoplasia. A prospective, randomized comparison between loop diathermy excision and laser excisional conization. Am J Obstet Gynecol 1993;168:485–8.
5. Luesley DM, Cullimore J, Redman CW, Lawton FG, Emens JM, Rollason TP, et al. Loop diathermy excision of the cervical transformation zone in patients with abnormal cervical smears. BMJ 1990;300:1690–3.
6. Wright TC Jr, Gagnon S, Richart RM, Ferenczy A. Treatment of cervical intraepithelial neoplasia by using the loop electrosurgical excision procedure. Obstet Gynecol 1992;79:173–8.
7. Sawaya GF, Brown AD, Washington AE, Garber AM. Clinical practice. Current approaches to cervical-cancer screening. N Engl J Med 2001;344:1603–7.
8. Benedet JL, Miller DM, Nickerson KG. Results of conservative management of cervical intraepithelial neoplasia. Obstet Gynecol 1992;79:105–10.
9. Townsend DE, Richart RM. Cryotherapy and carbon dioxide laser management of cervical intraepithelial neoplasia: a controlled comparison. Obstet Gynecol 1983;61:75–8.
10. Berget A, Andreasson B, Bock JE, Bostofte E, Hebjorn S, Isager-Sally L, et al. Outpatient treatment of cervical intra-epithelial neoplasia. The CO^2 laser versus cryotherapy, a randomized trial. Acta Obstet Gynecol Scand 1987;66:531–6.
11. McCrory DC, Matchar DB, Bastian L, Datta S, Hasselblad V, Hickey J, et al. Evaluation of cervical cytology. Evidence report/technology assessment no. 5. (Prepared by Duke University under Contract No. 290-97-0014.) AHCPR Publication No. 99-E010. Rockville, MD: Agency for Health Care Policy and Research. February 1999.
12. Myers ER, McCrory DC, Nanda K, Bastian L, Matchar DB. Mathematical model for the natural history of human papillomavirus infection and cervical carcinogenesis. Am J Epidemiol 2000;151:1158–71.
13. Bernstein AB, Thompson GB, Harlan LC. Differences in rates of cancer screening by usual source of medical care. Data from the 1987 National Health Interview Survey. Med Care 1991;29:196–209.
14. Ostor AG. Natural history of cervical intraepithelial neoplasia: a critical review. Int J Gynecol Pathol 1993;12:186–92.
15. Rice PS, Mant C, Cason J, Bible JM, Muir P, Kell B, et al. High prevalence of human papillomavirus type 16 infection among children. J Med Virol 2000;61:70–5.
16. Jacobs MV, Walboomers JM, Snijders PJ, Voorhorst FJ, Verheijen RH, Fransen-Daalmeijer N, et al. Distribution of 37 mucosotropic HPV types in women with cytologically normal cervical smears: the age-related patterns for high-risk and low-risk types. Int J Cancer 2000;87:221–7.
17. Richardson H, Franco E, Pintos J, Bergeron J, Arella M, Tellier P. Determinants of low-risk and high-risk cervical human papillomavirus infections in Montreal University students. Sex Transm Dis 2000;27:79–86.
18. Ho GY, Bierman R, Beardsley L, Chang CJ, Burk RD. Natural history of cervicovaginal papillomavirus infection in young women. N Engl J Med 1998;338:423–8.
19. Alexandrova YN, Lyshchov AA, Safronnikova NR, Imyanitov EN, Hanson KP. Features of HPV infection among the healthy attendants of gynecological practice in St. Petersburg, Russia. Cancer Lett 1999;145:43–8.
20. Moscicki AB, Shiboski S, Broering J, Powell K, Clayton L, Jay N, et al. The natural history of human papillomavirus infection as measured by repeated DNA testing in adolescent and young women. J Pediatr 1998;132:277–84.
21. Hildesheim A, Schiffman MH, Gravitt PE, Glass AG, Greer CE, Zhang T, et al. Persistence of type-specific human papillomavirus infection among cytologically normal women. J Infect Dis 1994;169:235–40.
22. Herrero R, Hildesheim A, Bratti C, Sherman ME, Hutchinson M, Morales J, et al. Population-based study of human papillomavirus infection and cervical neoplasia in rural Costa Rica. J Natl Cancer Inst 2000;92:464–74.

23. Liaw KL, Glass AG, Manos MM, Greer CE, Scott DR, Sherman M, et al. Detection of human papillomavirus DNA in cytologically normal women and subsequent cervical squamous intraepithelial lesions. J Natl Cancer Inst 1999;91:954–60.
24. Liaw KL, Hsing AW, Chen CJ, Schiffman MH, Zhang TY, Hsieh CY, et al. Human papillomavirus and cervical neoplasia: a case-control study in Taiwan. Int J Cancer 1995;62:565–71.
25. Schiffman MH, Bauer HM, Hoover RN, Glass AG, Cadell DM, Rush BB, et al. Epidemiologic evidence showing that human papillomavirus infection causes most cervical intraepithelial neoplasia. J Natl Cancer Inst 1993;85:958–64.
26. Bergeron C, Barrasso R, Beaudenon S, Flamant P, Croissant O, Orth G. Human papillomaviruses associated with cervical intraepithelial neoplasia. Great diversity and distinct distribution in low- and high-grade lesions. Am J Surg Pathol 1992;16:641–9.
27. Goldie SJ, Kuhn L, Denny L, Pollack A, Wright TC. Policy analysis of cervical cancer screening strategies in low-resource settings: clinical benefits and cost-effectiveness. JAMA 2001;285:3107–15.
28. Syrjanen K, Kataja V, Yliskoski M, Chang F, Syrjanen S, Saarikoski S. Natural history of cervical human papillomavirus lesions does not substantiate the biologic relevance of the Bethesda System. Obstet Gynecol 1992;79:675–82.
29. Holowaty P, Miller AB, Rohan T, To T. Natural history of dysplasia of the uterine cervix. J Natl Cancer Inst 1999;91:252–8.
30. Weaver MG, Abdul-Karim FW, Dale G, Sorensen K, Huang YT. Outcome in mild and moderate cervical dysplasias related to the presence of specific human papillomavirus types. Mod Pathol 1990;3:679–83.
31. Bibbo M, Dytch HE, Alenghat E, Bartels PH, Wied GL. DNA ploidy profiles as prognostic indicators in CIN lesions. Am J Clin Pathol 1989;92:261–5.
32. Luthra UK, Prabhakar AK, Seth P, Agarwal SS, Murthy NS, Bhatnagar P, et al. Natural history of precancerous and early cancerous lesions of the uterine cervix. Acta Cytol 1987;31:226–34.
33. Nasiell K, Roger V, Nasiell M. Behavior of mild cervical dysplasia during long-term follow-up. Obstet Gynecol 1986;67:665–9.
34. Lindgren J, Vesterinen E, Purola E, Wahlstrom T. Prognostic significance of tissue carcinoembryonic antigen in mild dysplasia of the uterine cervix. Tumour Biol 1986;6:465–70.
35. van Oortmarssen GJ, Habbema JD. Epidemiological evidence for age-dependent regression of pre-invasive cervical cancer. Br J Cancer 1991;64:559–65.
36. Campion MJ, McCance DJ, Cuzick J, Singer A. Progressive potential of mild cervical atypia: prospective cytological, colposcopic, and virological study. Lancet 1986;2:237–40.
37. Krahn M, Guasparini R, Sherman M, Detsky AS. Costs and cost-effectiveness of a universal, school-based hepatitis B vaccination program. Am J Public Health 1998;88:1638–44.
38. Deuson RR, Hoekstra EJ, Sedjo R, Bakker G, Melinkovich P, Daeke D, et al. The Denver school-based adolescent hepatitis B vaccination program: a cost analysis with risk simulation. Am J Public Health 1999;89:1722–7.
39. Kwan-Gett TS, Whitaker RC, Kemper KJ. A cost-effectiveness analysis of prevaccination testing for hepatitis B in adolescents and preadolescents. Arch Pediatr Adolesc Med 1994;148:915–20.
40. Margolis HS, Coleman PJ, Brown RE, Mast EE, Sheingold SH, Arevalo JA. Prevention of hepatitis B virus transmission by immunization. An economic analysis of current recommendations. JAMA 1995;274:1201–8.
41. Eddy DM. Screening for cervical cancer. Ann Intern Med 1990;113:214–26.
42. Brown AD, Garber AM. Cost-effectiveness of 3 methods to enhance the sensitivity of Papanicolaou testing [see comments]. JAMA 1999;281:347–53.
43. Wright TC, Sun XW, Koulos J. Comparison of management algorithms for the evaluation of women with low-grade cytologic abnormalities. Obstet Gynecol 1995;85:202–10.
44. Helms LJ, Melnikow J. Determining costs of health care services for cost-effectiveness analyses: the case of cervical cancer prevention and treatment. Med Care 1999;37:652–61.
45. Taylor LA, Sorensen SV, Ray NF, Halpern MT, Harper DM. Cost-effectiveness of the conventional papanicolaou test with a new adjunct to cytological screening for squamous cell carcinoma of the uterine cervix and its precursors. Arch Fam Med 2000;9:713–21.
46. Melnikow J, Nuovo J, Paliescheskey M. Management choices for patients with squamous atypia on Papanicolaou smear. A toss up? Med Care 1996;34:336–47.
47. Lepine LA, Hillis SD, Marchbanks PA, Koonin LM, Morrow B, Kieke BA, et al. Hysterectomy surveillance—United States, 1980–1993. MMWR CDC Surveill Summ 1997;46:1–15.
48. Institute of Medicine (U.S.). Committee to Study Priorities for Vaccine Development, Stratton KR, Durch J, Lawrence RS. Vaccines for the 21st century: a tool for

decisionmaking Washington: National Academy Press; 2000.

49. Fryback DG, Dasbach EJ, Klein R, Klein BE, Dorn N, Peterson K, et al. The Beaver Dam Health Outcomes Study: initial catalog of health-state quality factors. Med Decis Making 1993;13:89–102.
50. Gold MR. Cost-effectiveness in health and medicine. New York: Oxford University Press; 1996.
